# Detection of *Cutibacterium* (*Propionibacterium*) *acnes* in orthopaedic surgery: serious problem or contamination?

**DOI:** 10.1007/s00264-023-05981-w

**Published:** 2023-09-20

**Authors:** Christina Otto-Lambertz, Ayla Yagdiran, Alessa Lalinka Boschert, Peer Eysel, Sarah Victoria Walker

**Affiliations:** 1 Department of Orthopaedic Surgery, University of Cologne, Kerpener Straße 62, 50937 Cologne, Germany; 2 Institute for Medical Microbiology, Immunology and Hygiene, University of Cologne, Goldenfelsstraße 19-21, 50935 Cologne, Germany; 3 German Centre for Infection Research (DZIF), Hannover, Germany; 4 Institute for Clinical Microbiology and Hospital Hygiene, Hospital Ludwigsburg, Ludwigsburg, Germany

**Keywords:** Bone infection, *Cutibacterium acne*s, *Propionibacterium acnes*, Orthopedic surgery

## Abstract

**Purpose:**

Bone and joint infections are an important and increasing problem. Whether intraoperatively detected bacteria should be considered relevant or not is often difficult to assess.

This retrospective cohort study analyzes the relevance of *C. acnes* cultured from deep intraoperative specimens.

**Methods:**

All deep tissue samples collected intraoperatively between 2015 and 2020 from a quartiary care provider were evaluated for detection of *C. acnes* and its therapeutical consequences. Infection rates were determined according to a standardized definition and protocol and analyzed in dependence of patient’s demographic data (age and gender), operative parameters (type of surgery, body region/location of surgery, and impression of the surgeon), and initiated therapy.

**Results:**

In 270 cases of more than 8500 samples, *C. acnes* was detected. In 30%, the detection was considered an infection. The number of samples taken and tested positive for *C. acnes* correlated significantly with its classification as a cause of infection. If more than one sample of the patient was positive, the detection was significantly more likely to be treated as infection (*p* < 0.001).

In 76% of cases, a consultation to the infectious diseases (ID) department took place regarding the classification of the pathogen detection and the therapy to be carried out.

Almost all of the tested isolates demonstrated the wild-type susceptibility for penicillin and clindamycin.

**Conclusion:**

Intraoperative detection of skin-colonizing bacteria such as *C. acnes* is not always synonymous with infection. In particular, if other examination results contradict an infection (pathological sample without evidence of an infectious event, detection of malignant cells, etc.), the situation must be considered in a very differentiated manner. Interdisciplinary boards, for example, are suitable for this purpose. Care should be taken to obtain a sufficiently large number of tissue samples for microbiological examination to be able to better classify the result.

## Introduction

Resident skin bacteria such as *Cutibacterium* (*C.*) *acnes* (formerly *Propionibacterium* (*P.*) *acnes*) are regularly found among the microbiological specimen in postoperative infections of the bone and of the soft tissues [1, 2]. However, it often remains unclear whether the detection of *C. acnes* really reflects an infection or rather is merely an intraoperative contamination of the wound without the need for therapy.

*C. acnes* is the typical trigger of acne vulgaris, which is one of the most common skin diseases worldwide. It usually manifests between puberty and the age of 30 as an inflammatory disease of the skin appendages, primarily the sebaceous glands and hair follicles. Previously, the general recommendation for treatment of acne vulgaris was a combined systemic and topical therapy. Yet, due to this therapy, skin microbiota are under selective pressure resulting in antibiotic-resistant strains. Considering the global rise of multiresistant bacteria, the aforementioned therapy causes increasing criticism [3]. Therefore, a modification of the therapy recommendation, away from systemic antibiotic therapies, took place in recent years.

Apart from its role as an opportunistic pathogen in acne vulgaris [4], *C. acnes* has also been described in opportunistic infections of implants due to its ability to form biofilms [5, 6]. Biofilm formation is an important virulence factor that needs to be considered in treatment [7]. Rifampicin may be effective against *C. acnes* biofilms, but the data on this are not yet validated and are mainly based on experimental (animal) studies [8, 9].

Due to the increasing development of resistance, including that of *C. acnes*, it is relevant to differentiate under what conditions *C. acnes* detected in wounds is considered therapy-relevant, i.e., as the trigger of an infection, and when detection of *C. acnes* during surgery is not considered relevant and is, therefore, not treated. As the incidence of bone and joint infections is increasing worldwide and is associated with high mortality [10–12], the importance of *C. acnes* in orthopedic surgery is also becoming more relevant. In addition, the identification of resistant *C. acnes* strains is a matter of interest.

## Material and methods

All deep tissue samples taken intraoperatively during orthopaedic procedures (spine surgery, tumor surgery, joint surgery, and paediatric orthopaedic surgery) in a quartiary care hospital between 2015 and 2020 were retrospectively examined. The tissue specimen is routinely collected during intraoperative procedures involving bone or joints to evaluate/exclude infectious pathogenesis. Superficial samples such as wound and skin swabs were excluded from the study.

After removal, the samples were packaged sterile on the operating table and taken to the clinic’s microbiological institute, where the microbiological examination was carried out. Cultural diagnostics was performed in accordance to the German quality standards in microbiology. The examination of the samples referred to the detection of *C. acnes*. Antimicrobial resistance was routinely determined in accordance with EUCAST guidelines for penicillin and clindamycin but only occasionally for rifampicin. The incubation time of deep intraoperative samples was always 14 days.

If *C. acnes* was detected, the patient records were examined for the therapeutic consequences of the detection. Subsequently, the percentage of *C. acnes* positive samples in relation to the total of all microbiological samples taken per case was determined. Patient demographic data (age and gender), operative parameters (type of surgery, body region/location of surgery, and impression of the surgeon), and therapy factors were evaluated retrospectively.

According to the hospital’s internal guidelines, intraoperative detection of *C. acnes* in deep tissue samples was considered an infection if.

1. It was detectable in at least two out of several deep tissue samples taken intraoperatively
2. Histological examination of the tissue specimen showed definite evidence of osteomyelitis or acute soft tissue inflammation
3. The clinical findings and the surgeon’s assessment indicated an infection (clinical indicators, e.g., swelling of tissue/joint, hyperaemia, and/or pus)

Attending physicians could independently decide, whether an infection with *C. acnes* was present. Unclear cases were discussed together with the colleagues of the infectious diseases (ID) department (via bedside ID consultation or the in-house interdisciplinary board for bone and joint infections (osteomyelitis board, OMB)).

All data were extracted from the electronic health record and entered anonymously using Microsoft Excel 2010 software.

### Statistics

Counts and frequencies were used to describe the sample. All statistical operations are of an exploratory nature; therefore, no adjustments were made for multiple testing. The significance level was set at 5% for all reported inferential statistical operations. Data analyses and graphical depictions were performed using MSOffice.

## Results

**Fig. 1:**
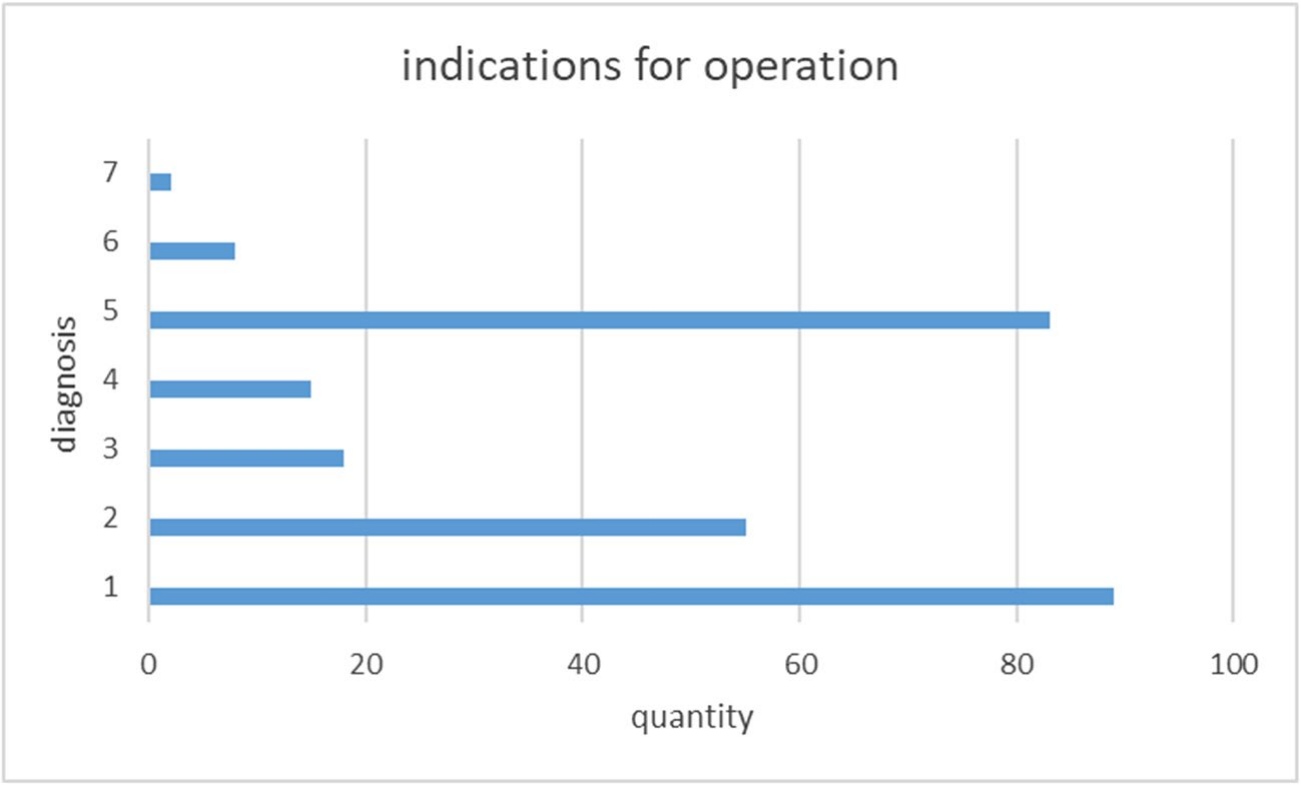
Indications for the operation of the patients with *Cutibacterium acnes* detection intraoperative: (1) spine surgery, (2) operation on an artificial joint, (3) operation on a native joint, (4) wound healing disorder post-operation, (5) tumor, (6) osteomyelitis, and (7) soft tissue abscess

During the six-year data collection period, more than 8,500 deep bone and tissue samples were collected intraoperatively from more than 1500 patients. *C. acnes* was detected at least once in 270 patients (indications for operation, see Fig. 1). 101 of the total number of patients were female, and 169 (62.6%) were male (Table 1). The mean age of the patients was 59 years (median (46–70 IQR)).

**Table 1:** Epidemic data of the included patients

Parameters	Number (%)
Number of patients with *Cutibacterium acnes* detection intraoperative	270
Male	169 (62.6%)
Age (years) (median (IQR)) total	59 (46–70)
Age (years) (median (IQR)) men	62 (48–71)
Age (years) (median (IQR)) women	55 (44–69)
Number of patients quoted as *Cutibacterium acnes* infection	81

In 81 of total of 270 patients (30%), the *C. acnes* detection was considered an infection and was treated.

46 patients (56.8%) were pure *C. acnes* infections. 35 of the 81 patients had polymicrobial infections, in which *C. acnes* was also considered relevant and included in the antimicrobial therapy/procedure.

Most of the patients ruled to have infection were male (59, 72.8%).

**Fig. 2:**
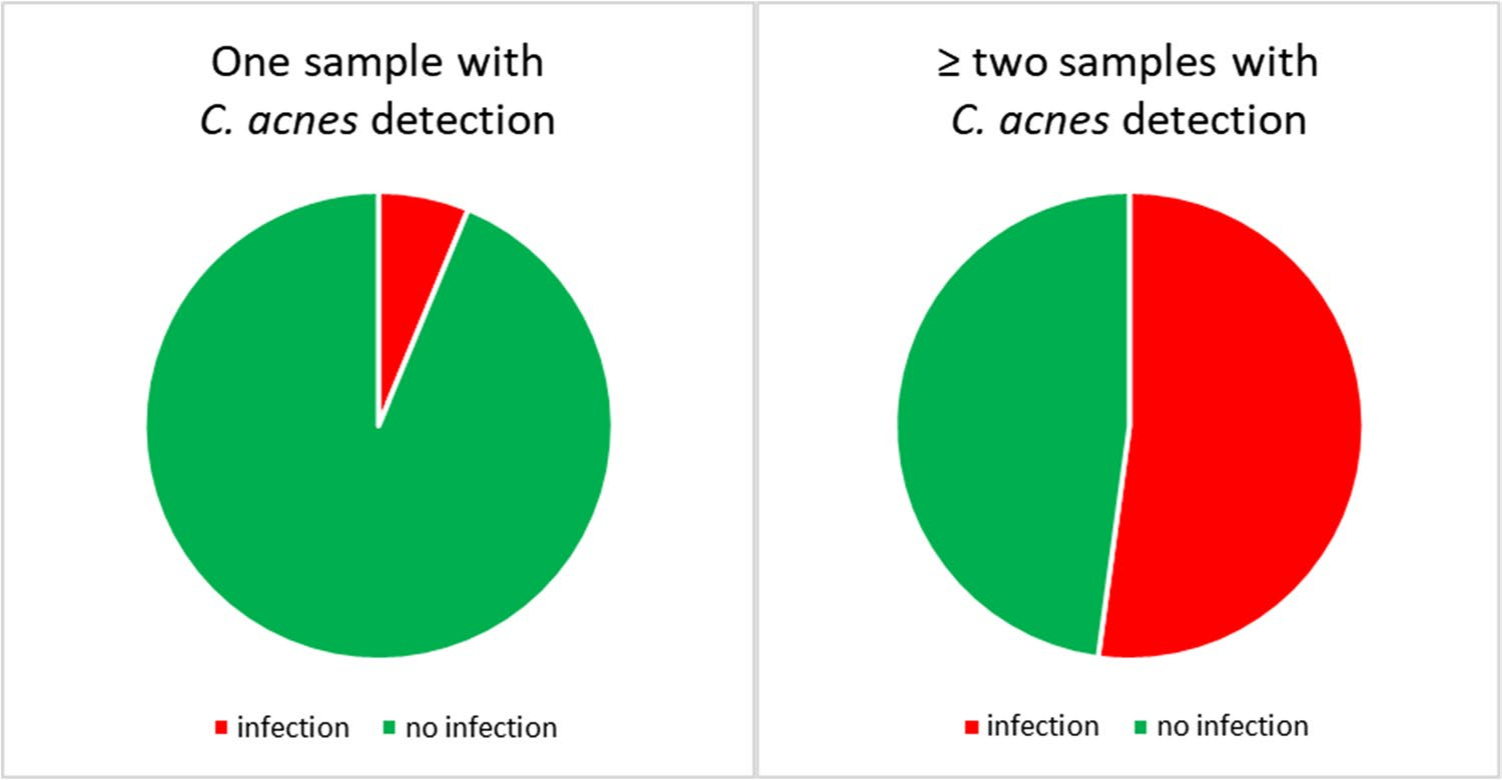
If more than one sample of the patient was positive for *C. acnes*, the detection was significantly more likely to be treated as infection (*p* = 0.001)

**Fig. 3:**
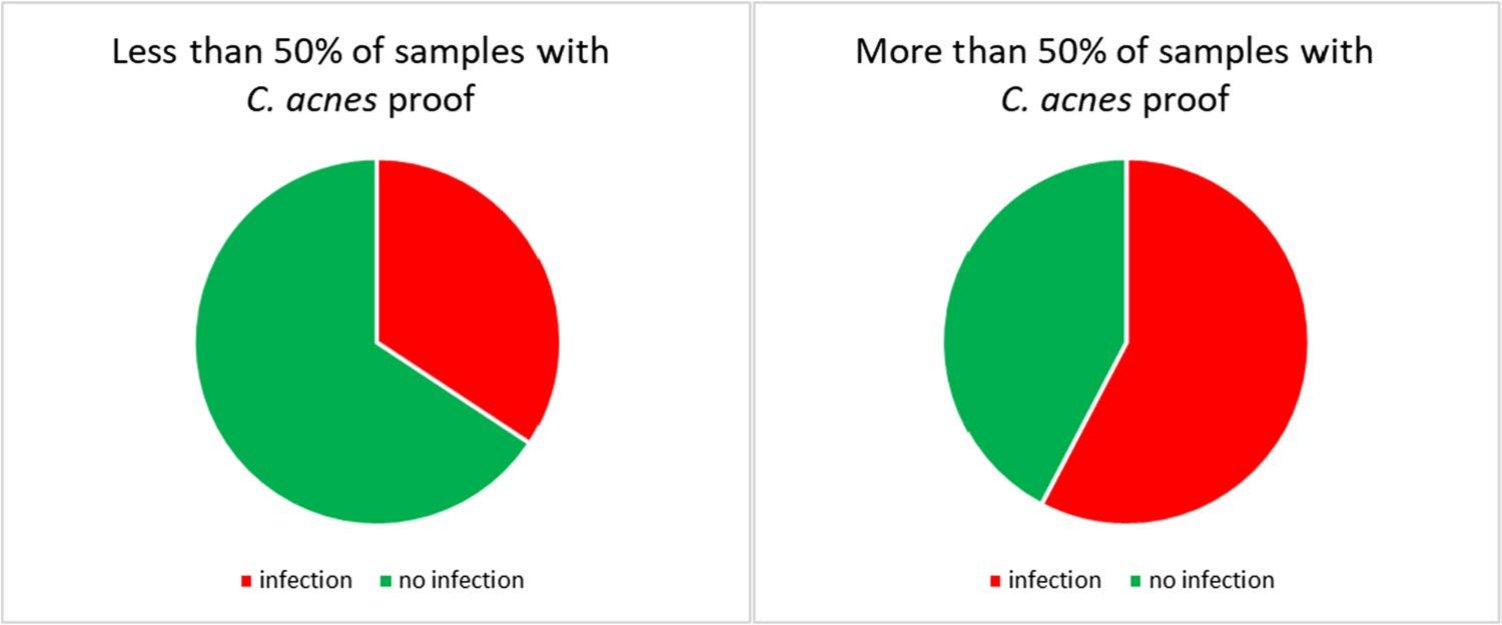
Multiple detection of *C. acnes* was significantly more likely to be an infection if more than 50% of the intraoperative specimens collected were positive for the pathogen (*p* < 0.05)

If more than one sample of the patient was positive, the detection was significantly more likely to be treated as infection (*p* = 0.001; Fig. 2). Furthermore, multiple detection of *C. acnes* was significantly more likely to be an infection if more than 50% of the intraoperative specimens collected were positive for the pathogen (*p* < 0.05) (Fig. 3).

In 47 of 81 patients (58%) where *C. acnes* detection was considered an infection, it was cultivated in all intraoperatively collected specimens of these patients. In 70 of the 81 patients (84.4%), *C. acnes* was cultivated in more than 50% of the intraoperatively collected specimens. In the 11 patients (13.6%) rated as “infected” with *C. acnes*, where the pathogen could only be detected in less than 50% of the samples, the decisive criterion that classified the case as an infection was the surgeon’s statement describing a purulent intraoperative situs.

In rare cases, the pathology specimen revealed evidence of osteomyelitis.

In pure *C. acnes* infections, penicillin G was used for intravenous therapy in 37% (17 ×) for two weeks, followed by oral antibiotic therapy with clindamycin (40 × , 87%; alternatively amoxicillin 3 × , amoxicillin-clavulanic acid 1 × , levofloxacin 1 × , or doxycycline 1 ×). The total duration of therapy was usually six to 12 weeks.

In 62 of the infectious patients (76.5%), an ID “consultation” took place regarding the classification of the pathogen detection and the therapy to be carried out. 64.2% of these patients were advised by a bedside ID consultation, 37% by the clinic’s OMB. In 21 patients (25.9%), the case was discussed both (ID consultation and OMB).

**Fig. 4:**
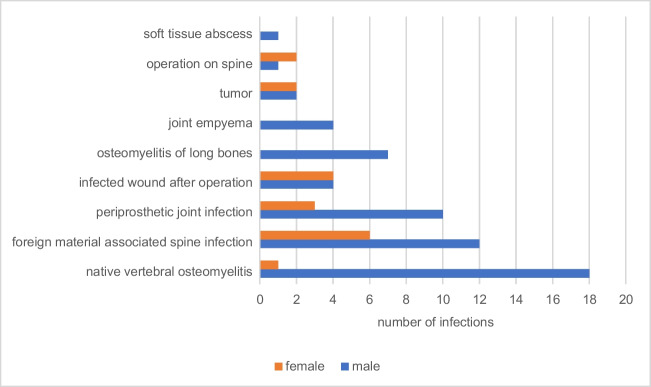
The most common diagnosis of the 81 patients evaluated as infection

The most common diagnosis in patients evaluated as infection was spondylodiscitis (19 × ; 23.5%), followed by foreign material-associated infection of the spine (18 × ; 22.2%). Less common was total joint arthroplasty infection (13 × ; 16%), postoperative infected wound healing disorders (8 × ; 9.9%), osteomyelitis of the extremity (6 × ; 7.4%), and joint empyema (4 × ; 4.9%) (Fig. 4).

In 46 cases (56.8%) of the 81 patients, the surgeon considered the situs to be “certainly infected” (macroscopic pus and turbid secretion). Pure *C. acnes* infections (25 cases) and polymicrobial infections (21 cases) balanced each other. In 40 of the 46 cases, *C. acnes* detection was successful collected in more than half of the samples.

In 82 cases out of the total number of 270 cases, the situs was purulent or considered certainly infected by the surgeon. Of these 82 patients, *C. acnes* detection was not considered an infection-causing pathogen in 30 patients, but other pathogens were detected (polymicrobial flora). In eight of the 82 patients, the detected *C. acnes* was not evaluated as an infectious pathogen, but no other pathogen was found in the incubation (macroscopic pus without relevant pathogen detection).

In 82 patients of the 270 patients, *C. acnes* was detected in “tumor”-associated operations. A lot of them were operations with sampling for clinically and radiologically unclear, malignant-impressive changes. Of these 82 patients, osteomyelitis was diagnosed in only one case by pathologic examination of the intraoperative specimen.

Of the remaining 81 patients, only four *C. acnes* detections were considered as an infection and treated, although in 59 patients of these 81, *C. acnes* detection occurred in at least half of the specimens, and in some cases in all specimens (47 cases). However, in 25 of the 81 patients, only one or two samples were taken, contrary to the general recommendation to take at least three, preferably five samples.

In only two of the 81 patients, the situs was intra-operatively assessed to be “certainly infectious” (see above).

The sampling method was mostly via a Yamshidi needle (bone punch) or open via luer/scalpel. An indication of whether, for example, a fresh/clean luer was used to take the samples was found in only a few cases.

In none of the patients with intraoperative *C. acnes* detection was there any mention of previous (or current) acne vulgaris therapy in the patient records reviewed and evaluated.

In 146 of all 270 cases, the interpretation of the examination results was purely by the treating orthopedist. In 124 cases (45.9%), ID consultation was performed, sometimes more than once. Of these, only bedside ID consultation was performed in 102 cases, and only OMB consultation was performed in 67 cases. In 46 cases, both (bedside ID consultation and OMB consultation) were performed.

### Resistance

All of the tested isolates (100%) demonstrated the wild-type susceptibility for penicillin and 96.4% of isolates were susceptible to clindamycin. Rifampicin was tested in 65 isolates and moxifloxacin in two isolates of which all of them demonstrated low minimum inhibitory concentrations (MIC) indicating potential likelihood of therapeutic success.

## Discussion

We present a comprehensive analysis of orthopedic infection patients caused by *C. acnes* from a retrospective cohort focusing on clinical characteristics. Our main findings were as follows:

(i) Detection of *C. acnes* was deemed a relevant infection requiring treatment in 30% of all cases (81 patients)
(ii) The number of samples taken and tested positive for *C. acnes* correlated significantly with its classification as a cause of infection
(iii) If more than one sample of the patient was positive, the detection was significantly more likely to be treated as infection (*p* < 0.001)7

From this retrospective evaluation of *C. acnes* detections from orthopaedic patients of a quartiary care provider, it is evident that not every detection of *C. acnes* is synonymous with infection by *C. acnes*, as is already known from studies, particularly in the field of shoulder surgery [13]. A similar result regarding the detection of *C. acnes* in blood cultures shows the work of Boman et al. [14].

The generally accepted recommendations (detection in more than one specimen, result of the additional pathological specimen if applicable, clinical impression, etc.) were used as decisive tool for evaluation of microbial detection. However, since result interpretation can be difficult, in 45.9% of all cases, an ID specialist was consulted. This was mostly done via the consultation service, alternatively via the hospital’s own board for bone and joint infections. The high percentage of cases in which both bedside ID consultation and presentation via the OMB occurred underlines the importance of interdisciplinary case discussions. Both “modes of presentation” have their own advantages and disadvantages. In bedside ID consultation, an ID specialist also clinically assesses the patient. However, the infectious disease recommendation is then usually determined without discussion of the case with the treating orthopaedic department, or even the surgeon. In contrast, there is no clinical co-examination of the patient by the ID department during the OMB. However, here, the case is discussed with all its facets with all the specialists involved (treating orthopedic ward physician, surgeon, infectiologist, radiologist/microbiologist/vascular surgeon/pathologist, etc.). Especially in ambiguous constellations, this joint discussion of the case is essential to be able to decide whether it is really an infection and whether (or which) therapy should be initiated. This result is consistent with the results reported in the literature. A recent study was able to show that interdisciplinary discussion of infection patients led in a high percentage to a change in therapy, or that additional diagnostics were recommended as an alternative [15].

According to the increasing numbers [16, 17], vertebral osteomyelitis was the most common among infectious patients.

The high number of tumor patients in the total quantity is due to the fact that the distinction between a (malignant) tumour disease and an infection of the bone is often difficult to make, clinically and radiologically. Basically, the high number of patients in whom *C. acnes* was detected in, partially in even all tissue samples taken, is striking. This microbiological detection of *C. acnes* was apparently ignored in cases of a clear pathological sample (either evidence of tumour tissue or at least pathological without evidence of florid infection of the tissue taken). No therapy of the pathogen was performed in all these cases, without a necessary new surgical intervention due to (progressive) infection evident from the files.

Considering the many tumour patients where *C. acnes* was cultivated in multiple samples, the question must be discussed (again) to what extent a separate, fresh/clean “collection device” should be used for each sample when samples are taken intraoperatively for microbiological examination. When using one device for multiple samples, there is of course an increased risk of spreading contamination of the bacterium from the skin to the sample via the device. However, literature offers little data on a general recommendation to change the collection device after each sample taken. Nevertheless, as a “knock-off,” similar to the Maki technique, it seems quite possible that pathogen spread via the instrument takes place, so that several positive samples with microbiological pathogen growth need not reflect a realistic situation. To better distinguish false-positive samples from true-positive samples, a defined “sampling protocol” of the samples documenting the sampling specifics in detail (place of sampling, instrument used, etc.) may be helpful. This procedure is currently recommended by some literature [18, 19].

Interestingly, there was a correlation between the clinical intraoperative aspect of the surgeon and the detection of bacteria. According to our data, the surgeon considered the intraoperative site to be definitely infected or purulent in only 56.8% of cases of *C. acnes* infection. In a high percentage of cases in which *C. acnes* was not judged to be an infection-causing pathogen, other pathogens (*Staphylococcus aureus*, etc.) were detected. This may be explained by the fact that *C. acnes* is not the typical pus-causing pathogen. Our data are in line with this finding. Nevertheless, it stresses the high relevance of the surgeon’s impression, since macroscopic detection of pus correlates well overall with general pathogen detection. Only in eight of 270 patients did the surgeon assess the situs as infected without bacteria being detected in the microbiological specimens.

### Resistance

Since 100% of the cases were of wild-type MIC distribution for penicillin and rifampicin and 96.4% for clindamycin, the data examined here do not indicate any problems concerning increased development of *C. acnes* resistance in bone and joint surgery. However, none of the patients affected or examined had a medical history of treatment for acne vulgaris. It would be of interest to investigate patients undergoing acne therapy in the past. This is planned as a prospective follow-up project.

### Limitations

This is a retrospective single center study. It is a matter of very heterogeneous data generated from different collection sites in the body, so the conclusions are therefore limited.

The results cannot be easily generalized and need to be validated for primary care hospitals and other countries.

Nevertheless, the data reflect some very interesting aspects of clinical practice with the recurrent debate of whether bacterial detection from deep tissue samples intraoperatively equates to infection of the situs. We would like to answer this question here clearly with no. As is already known from the field of shoulder surgery in particular, this statement can also be made across the entire spectrum of orthopaedic surgery, as we were able to show here. Intraoperative detection of skin-colonizing bacteria such as *C. acnes* is not always synonymous with infection. In particular, if other examination results contradict an infection (pathological sample without evidence of an infectious event, detection of malignant cells, etc.), the situation must be considered in a very differentiated manner. Interdisciplinary boards, for example, are suitable for this purpose. Care should be taken to obtain a sufficiently large number of tissue samples for microbiological examination in order to be able to better classify the result. The relevant guidelines provide instructions for this [20–27].

In addition, the resistograms of the cultivated *C. acnes* do not show any mutations or development of resistance. While it is not possible to be certain from the available retrospective data whether there are patients in this cohort who have undergone antibiotic therapy for acne vulgaris in the past, at least none of the patients reported such therapy in their medical history. However, further prospective surveys are still needed to disprove an increasing development of resistance of *C. acnes* by passing acne vulgaris therapy.

In conclusion, it can be said that the detection of *C. acnes* in deep tissue samples taken intraoperatively can and should only be assessed in conjunction with the patient’s clinical condition, the number of positive samples, and the intraoperative aspect of the surgeon.

## Data Availability

The original data is held by the corresponding author and can be viewed there at any time.
